# Influence of vascular endothelial growth factor stimulation and serum deprivation on gene activation patterns of human adipose tissue-derived stromal cells

**DOI:** 10.1186/s13287-015-0062-9

**Published:** 2015-04-13

**Authors:** Josefine Tratwal, Anders Bruun Mathiasen, Morten Juhl, Sonja Kim Brorsen, Jens Kastrup, Annette Ekblond

**Affiliations:** Cardiology Stem Cell Centre, The Heart Centre, Rigshospitalet, University Hospital Copenhagen, Juliane Maries Vej 20, dept. 9302, Copenhagen, 2100 Denmark

## Abstract

**Introduction:**

Stimulation of mesenchymal stromal cells and adipose tissue-derived stromal cells (ASCs) with vascular endothelial growth factor (VEGF) has been used in multiple animal studies and clinical trials for regenerative purposes. VEGF stimulation is believed to promote angiogenesis and VEGF stimulation is usually performed under serum deprivation. Potential regenerative molecular mechanisms are numerous and the role of contributing factors is uncertain. The aim of the current study was to investigate the effect of *in vitro* serum deprivation and VEGF stimulation on gene expression patterns of ASCs.

**Methods:**

Gene expressions of ASCs cultured in complete medium, ASCs cultured in serum-deprived medium and ASCs stimulated with VEGF in serum-deprived medium were compared. ASC characteristics according to criteria set by the International Society of Cellular Therapy were confirmed by flow cytometry. Microarray gene expressions were obtained using the Affymetrix HT HG-U133+ GeneChip®. Gene set enrichment analysis was performed using the Kyoto Encyclopedia of Genes and Genomes and gene ontology terms. Transcription of selected genes of interest was confirmed by quantitative PCR.

**Results:**

Compared to ASCs in complete medium, 190 and 108 genes were significantly altered by serum deprivation and serum deprivation combined with VEGF, respectively. No significant differences in gene expression patterns between serum-deprived ASCs and serum-deprived ASCs combined with VEGF stimulation were found. Genes most prominently and significantly upregulated by both conditions were growth factors (*IGF1, BMP6, PDGFD, FGF9*), adhesion molecule *CLSTN2*, extracellular matrix-related proteins such as matricellular proteins *SMOC2*, *SPON1* and *ADAMTS12*, and inhibitors of proliferation (JAG1). The most significantly downregulated genes included matrix metalloproteinases (*MMP3*, *MMP1*), and proliferation markers (*CDKN3*) and *GREM2* (a *BMP6* antagonist).

**Conclusion:**

The decisive factor for the observed change in ASC gene expression proves to be serum starvation rather than VEGF stimulation. Changes in expression of growth factors, matricellular proteins and matrix metalloproteinases in concert, diverge from direct pro-angiogenic paracrine mechanisms as a primary consequence of the used protocol. *In vitro* serum starvation (with or without VEGF present) appears to favour cardioprotection, extracellular matrix remodelling and blood vessel maturation relevant for the late maturation phase in infarct healing.

## Introduction

Adipose tissue-derived stromal cells (ASCs) are being clinically tested as regenerative therapy for patients with ischaemic heart disease to improve myocardial perfusion and to regenerate injured myocardium [1]. Though the detailed mechanisms of action are as of yet not completely defined, the outcome of ASC therapy is promoted by the vasculogenic or angiogenic abilities. Aiming to enhance the efficacy of stem cell therapy, the cells may be preconditioned with various substances to prime them into more specialised functional patterns prior to administration.

One of the most widely used preconditioning substances so far is vascular endothelial growth factor A-165 (VEGF). Early *in vitro* and pre-clinical studies suggested that VEGF, traditionally in combination with serum deprivation, facilitated differentiation of mesenchymal stromal cells (MSCs) and ASCs in an endothelial direction [2-4], whereas current thinking favours the notion of activation of resident cells through secretion of cytokines and growth factors [5]. Potential regenerative molecular mechanisms are, however, numerous and the role of contributing factors is still uncertain. On this basis, VEGF stimulation and serum deprivation of MSCs has been used in clinical studies including the double-blind, placebo-controlled trial MyStromalCell, which treats patients with chronic ischemic heart disease with VEGF pre-stimulated and serum-deprived ASCs [1,6,7].

In a recent study, we found a subtle priming only of ASC differentiation in an endothelial direction when ASCs were preconditioned with VEGF and serum deprivation compared to standard culturing conditions [8]. This raised the question whether VEGF preconditioning and serum deprivation of ASCs induces angiogenesis on a paracrine level by stimulating endothelial cells and perhaps also induces modifications in extracellular tissue components to facilitate improved microcirculation. As such, it is of great interest to elucidate the molecular signature of ASCs *per se* and of ASCs that have been preconditioned with VEGF and serum deprivation to compare their constitutive expression patterns with regard to angiogenesis and other tissue regenerative or stem cell-associated properties.

The aim of this study was to evaluate the gene expression profile of ASCs preconditioned with VEGF and/or serum deprivation using culture conditions similar to those applied in clinical stem cell trials [1]. With microarray analysis of ASCs under these conditions it is possible to evaluate changes in gene expression in genes of importance for tissue reconstruction and angiogenesis. We highlight the genes of relevance for angiogenesis as VEGF is believed to promote neovascularisation.

## Methods

### Donors

Lipoaspirate was obtained from eight healthy donors (two male and six female; mean age 41.5 years, range 21 to 57 years). All participants signed an informed consent. This complied with the declaration of Helsinki and the study was approved by the Ethical Committee, The Capital Region of Denmark protocol no. H-3-2009-119.

### Lipoaspirate preparation

Approximately 100 ml lipoaspirate was obtained from each participant from the abdomen by liposuction under local anaesthesia. The lipoaspirate was washed twice with phosphate-buffered saline (PBS) pH 7.4 (GIBCO, Life Technologies, Paisley, UK) to remove residual blood. The adipose tissue was digested by incubation with Collagenase NB 4 (SERVA Electrophoresis GmbH, Heidelberg, Germany) dissolved in HBSS (2 mM Ca^2+^, GIBCO, Life Technologies) at 37°C for 45 minutes under constant rotation. The collagenase was neutralized with complete medium (Dulbecco’s modified Eagle's medium (DMEM), low glucose 1 g/l supplemented with 25 mM HEPES and L-Glutamin (GIBCO, Life Technologies), 10% fetal bovine serum pharma grade (FBS; GIBCO, Life Technologies), 1% penicillin/streptomycin (GIBCO, Life Technologies)) and filtered through a 100-μm mesh (Cell Strainer, BD Bioscience, San Jose, CA, USA). The remaining cells were centrifuged at 1,200 g for 10 minutes in RNase free tubes (BD Biosciences) at room temperature, re-suspended and counted using NucleoCounter® NC-100™ (Chemometec, Allerød, Denmark) according to the manufacturer’s instructions.

### Adipose tissue-derived stromal cell isolation and culture

Primary cell cultures of mononuclear cells were established by seeding 4.5 × 10^6^ cells/T75-flask (Nunc, Thermo Scientific, Roskilde, Denmark) in complete medium. The cells were incubated in standard conditions at 37°C in humid air with 5% CO_2_. After two days in culture, cells were washed with PBS (GIBCO, Life Technologies) to remove non‐adhering cells, and thereafter complete medium was added and changed every 3 to 4 days.

After approximately 1 week in culture, cells were detached with 3 ml TrypLE® (TrypLE® Select, Gibco) for 10 minutes at 37°C and neutralized with 7 ml complete medium and centrifuged at 300 g for 5 minutes, resuspended, counted on NucleoCounter®, frozen 1 × 10^6^ cells/1 ml FBS with 5% DMSO (WAK-Chemie Medical GmbH, Steinbach, Germany) at −80°C in Nalgene® Mr.Frosty freezing container (Sigma- Aldrich, St. Louis, MO, US) and transferred to liquid nitrogen the following day for storage. When initiating an experiment, ASCs at passage 1 were rapidly thawed and seeded in T75-flasks with media changed the following day. When the cultures reached 80% confluence, cells were passaged at 3 × 10^5^ cells/T75-flask. ASC characteristics were ascertained by flow cytometry according to The International Society for Cellular Therapy minimal criteria for defining multipotent mesenchymal stromal cells [9,10] (as described previously by Follin and colleagues, 2013 [8]).

### Serum deprivation and vascular endothelial growth factor stimulation

Stimulation of cells followed a clinical protocol as previously described [1,8]. ASCs at passage 2 were cultured in complete medium until 80% confluence, after which medium was changed to either: 1) complete medium; 2) serum‐deprived medium (DMEM with 2% FBS and 1% penicillin/streptomycin (GIBCO, Life Technologies)); or 3) VEGF stimulation medium consisting of serum‐deprived medium with 50 ng/ml recombinant human VEGF‐A_165_ (rhVEGFA_165_, R&D Systems, Minneapolis, MN, US). Cells were cultured for 1 week, and test media was renewed every 3 days, after which they were harvested for further processing.

### Nucleic acid extraction

After 1 week of cultivation in the three different media cells were washed with PBS (GIBCO, Life Technologies), detached with TrypLE® Select (GIBCO, Life Technologies), and centrifuged for 5 minutes at 300 g. Then 350 μl lysis buffer was added to the cell pellet from the Qiagen RNeasy® Mini Kit (QIAGEN Hamburg GmbH, Hamburg, Germany) and a 1 ml syringe (B. Braun Melsungen AG, Melsungen, Germany) was used to mechanically lyse the cells before resuming the Qiagen protocol. Finally, total RNA was eluted with RNase-free water (5 Prime GmbH, Hamburg, Germany). RNA purity and concentration was measured using a NanoDrop® 1000 Spectrophotometer (Thermo Scientific, Waltham, MA, US), and the eluate was stored at –80°C until further analyses. RNA purity was validated by absorbance ratios at 260 nm/280 nm and protein contamination at A260/230. RNA integrity was confirmed by RIN >8 using RNA Nano Chips (Agilent Technologies, Santa Clara, CA, US) for the Agilent 2100 Bioanalyzer.

### Microarray

RNA samples, eight from each of the three cultivation conditions, were thawed and a final concentration of 33 ng/μl total RNA per sample was used for microarray analysis. Samples were analysed separately. Expression profiling was achieved using the Affymetrix GeneChip® HT HG-U133+ 24-Array Plate (Affymetrix, Santa Clara, CA, USA). Samples were labelled with Affymetrix Sensation Plus kit (Affymetrix) with 50 ng total RNA as starting material. Biotin labelled cDNA (4 μg) was used for the hybridization cocktail, and hybridization, washing, staining and scanning were performed with an Affymetrix Gene Titan instrument (Affymetrix). Microarray data are available in the ArrayExpress database [11] under accession number E-MTAB-3066.

### Microarray analysis

Microarray analysis was performed using R 3.01 [12] and Bioconductor [13]. The following gene expression comparisons were made: 1) ASCs from serum deprived medium versus ASCs from complete medium; 2) ASCs from serum deprived medium versus ASCs from serum-deprived medium stimulated with VEGF; and 3) ASCs from complete medium versus ASCs from serum-deprived medium stimulated with VEGF.

For each comparison the CEL files were imported into R/Bioconductor and RMA (Robust Multi-array Average) normalized. After this, all gene probes with a log2-fold change below 0.5 were omitted. The expression levels of each remaining gene were compared between the groups using paired *t*-tests. *P*-values were adjusted using the Benjamini and Hochberg procedure for multiple testing corrections. Log2-fold change values were calculated for each gene. Gene set enrichment analyses were made using Webgestalt [14] for enriched gene-sets in gene ontology (GO) categories and in enriched Kyoto Encyclopedia of Genes and Genomes (KEGG) categories.

### Genes of interest

Selection of gene categories of interest was partly explorative and partly predetermined. The different approaches involved were: 1) definition of groups using Gene Enrichment Analysis linked with KEGG library and GO terms as guiding templates for identifying unpredicted and relevant up- and downregulated gene categories from the whole human genome analysis; and 2) definition of predetermined categories relevant for expected response mechanisms. Predetermined categories include angiogenesis, proliferation, and secretome.

Single genes of interest were selected based on an elimination process of those genes that were at least two times significantly (*P* < 0.05) up- or downregulated corresponding to a fold change >0.9. Three of the most highly up- and downregulated genes were chosen for confirmatory quantitative PCR.

### Reverse transcription

The cDNA synthesis from total RNA was prepared using AffinityScript (Stratagene, Agilent Technologies) in a fast eight‐tube strip (0.1 ml, MicroAmp™, Applied Biosystems®, Life Technologies, Paisley, UK) on ice. The total reaction volume of 20 μl contained 0.5 μg RNA, 10 μl cDNA synthesis master mix, 3 μl Oligo dT primer, 1 μl AffinityScript RT RNase block enzyme mixture, and RNAase-DNAse free water (5 Prime GmbH) to 20 μl total volume. Reactions were performed with an initial stage of 25°C for 5 minutes, 42°C for 45 minutes, and 95°C for 5 minutes (Veriti 96 well fast thermal cycler, Applied Biosystems®, Life Technologies). Following synthesis, cDNA was stored in aliquots at –20°C.

### Confirmation by quantitative real-time PCR

Brilliant II SYBR®Green QPCR master mix with low ROX (Agilent Technologies) was used with a total reaction volume of 25 μl in 96-well optical reaction plates (Agilent Technologies) with 5 μl cDNA diluted 1:5 in 1 × EDTA (QIAGEN Hamburg GmbH, Hamburg, Germany) and subsequently 1:5 in RNAase-DNAse free water (5 Prime GmbH). The plates were sealed with optical plastic caps (Agilent Technologies). Quantitative PCR was performed with Mx3000 (Stratagene, AH-diagnostics, Aarhus, Denmark) and the results were collected using Mx3000 version 4.0 software for Windows (Stratagene, AH-diagnostics). The reaction was initiated by heating to 95°C for 10 minutes, followed by 40 cycles of elongation at 60°C for 1 minute and denaturation at 95°C for 30 seconds. Primers used are shown in Table 1.

**Table 1:** **Reference genes and genes of interest for confirmational quantitative PCR**

**Gene**	**Accession no.**	**Forward (sense) 5′→3′**	**Reverse (anti-sense) 5′→3′**	**Size, bp**
*SMOC2*	NM_022138	TTAAGGAACCATTTGGAGGACAG	CCACAAGCATCACAACATCAC	153
*BMP6*	NM_001718	CGTGAAGGCAATGCTCACCT	CCTGTGGCGTGGTATGCTGT	135
*IGF1*	NM_001111285	ACCGACATGCCCAAGACCCA	TTCAGCATTTCTACTTCCAATCTCCCT	185
*MMP1*	NM_001145938.1	GATGGACCTGGAGGAAATC	GTCCAAGAGAATGGCCGA	403
*MMP3*	NM_002422	CTGTTGATTCTGCTGTTGAG	AAGTCTCCATGTTCTCTAACTG	126
*ITGB3*	NM_000212	GGACACAGCCAACAACCCAC	AGGAGGCATTCTGGGACAAAG	348
*TBP*	NM_003194.4	TGCACAGGAGCCAAGAGTGAA	CACATCACAGCTCCCCACCA	339
*YWHAZ*	NM_001135702.1	ACTTTTGGTACATTGTGGCTTCAA	CCGCCAGGACAAACCAGTAT	245

Regulated genes of interest selected for confirmational quantitative PCR including reference genes selected with consideration to donor- and treatment-variation. Table presents full name, NCBI accession number and forward and reverse primer sequences.

### Quantitative PCR analysis

Reference gene(s) for the quantitative PCR were selected for the experimental conditions from a larger panel as described previously [15] by taking into account effect of donor- and treatment-variation on the reference genes. Comparisons were made by the GenEx software (MultiD Analyses AB, Goeteborg, Sweden) with the subprograms geNorm and Normfinder. *TATA-binding protein (TBP)* and *Tyrosine 3/tryptophan 5-monooxygenase activation protein* (*YWHAZ*) in combination were the most suitable reference genes for this set-up (Table 1). ΔΔCq normalization of quantitative PCR data with 2^ΔΔCq^ as the fold change was used. Normalized data were analysed in SPSS (Statistical Package for the Social Sciences IBM Denmark, Holte, Denmark) with a paired *t*-test for significant differences with a 95% confidence interval.

## Results

### Microarray analysis

#### Adipose tissue-derived stromal cells from complete medium compared to adipose tissue-derived stromal cells from serum-deprived medium

A total of 615 probes, corresponding to 190 genes, were found to be significantly up- or downregulated (*P* < 0.05).

#### Adipose tissue-derived stromal cells from complete medium compared to adipose tissue-derived stromal cells from serum-deprived medium stimulated with vascular endothelial growth factor

The comparison demonstrated that 304 probes corresponding to 108 genes differed significantly.

#### Adipose tissue-derived stromal cells from serum-deprived medium compared to adipose tissue-derived stromal cells from serum-deprived medium stimulated with vascular endothelial growth factor

No significant differences were observed between the two groups. An insignificant tendency was, however, seen for a slightly greater fold change of genes expressed in VEGF-stimulated ASCs than ASCs from serum-deprived medium compared to ASCs from complete medium. As a consequence, of the lack of statistical differences in regulation of genes between serum-deprived and VEGF-stimulated ASCs, final comparisons were made between serum-deprived or VEGF-stimulated ASCs versus control ASCs in complete medium only.

Some of the genes selected below only reached significance in one of the two test conditions compared to control cultures. Common to those genes is that regulation moved in the same direction for both test conditions but only reached a significant level in one.

### Selection of genes of interest

**Figure 1:**
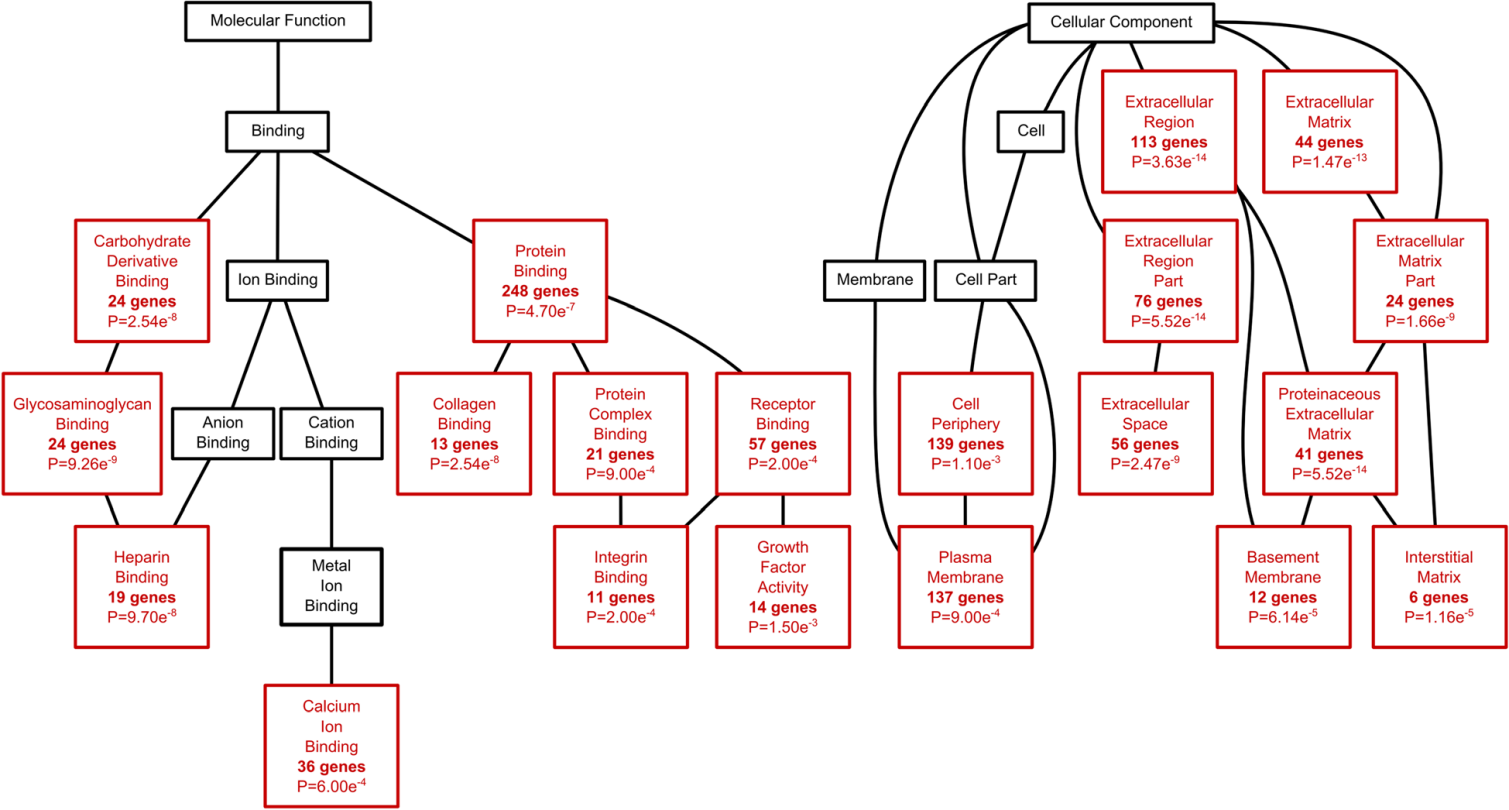
Directed acyclic graphs. Directed acyclic graphs (DAG) showed a strong representation of regulated genes employing proteins in the extracellular space particularly within the categories extracellular matrix and protein binding comprising integrin binding and growth factor activity (section of gene ontology analysis top 10 significantly regulated genes; serum deprivation compared to 10% serum in culture medium).

Gene Enrichment Analysis linked with KEGG library and GO terms and directed acyclic graphs showed a strong representation of regulated genes from the extracellular region, particularly within the categories “extracellular matrix” and “regulation of cell adhesion” (Figure 1). Consequently, categories “adhesion” and “extracellular matrix” were included as supplements to pre-determined categories “angiogenesis”, “proliferation”, and “secretome”. Significantly up- or downregulated genes, representing chosen categories, as determined by GO terms, were selected (Tables 2 and 3).

**Table 2:** **Expression levels for regulated genes according to primary gene ontology function**

		**Serum-deprived**			**VEGF-stimulated**		
**Gene ID**	**Gene name**	**FC**	**2** ^**|FC|**^		**FC**	**2** ^**|FC|**^	
***Adhesion***							
*CLSTN2*	Calsyntenin 2	+2.375		+5.187	+2.456		+5.488
*JAM2*	Junctional adhesion molecule B	+1.079		+2.112	NS		NS
*ITGB3*	Integrin, beta 3	*−0.709*		*−1.635*	−1.040		−2.056
*PODXL*	Podocalyxin-like protein 1	−1.126		−2.183	−1.066		−2.093
***Extracellular matrix***							
*SMOC2*	SPARC related modular calcium binding 2	+2.059		+4.167	+2.185		+4.548
*SPON1*	Spondin 1, F-spondin, extracellular matrix protein	+1.120		+2.174	+1.600		+3.039
*ADAMTS12*	ADAM metallopeptidase with thrombospondin type 1 motif	+1.136		+2.198	+1.213		+2.318
*SGCD*	Sarcoglycan delta	+1.027		+2.038	*+0.989*		*+1.985*
*DPT*	Dermatopontin	*+0.978*		+1.970	+1.006		+2.008
*MFAP4*	Microfibrillar-associated protein 4	+0.950		+1.933	+0.964		+1.951
*PTPRB*	Protein tyrosine phosphatase, receptor type, B	NS		NS	−0.9992		−1.999
*TGM2*	Transglutaminase 2	−1.063		−2.090	NS		NS
*MMP3*	Matrix metallopeptidase 3	−1.493		−2.816	−1.499		−2.826
*COL13A1*	Collagen, type XIII, alpha 1	−1.1227		−2.178	−0.9474		−1.028
*MMP1*	Matrix metallopeptidase 1	−2.139		−4.405	NS		NS

Groups defined by Gene Enrichment Analysis linked with Kyoto Encyclopedia of Genes and Genomes library and gene ontology terms. Fold changes (FC) and consequently calculated amount of up (+) or down (−) regulation (2^|FC|^) of significant genes (*P* < 0.05) compared to adipose tissue-derived stromal cells (ASCs) in complete medium are listed for vascular endothelial growth factor (VEGF)-treated and serum-deprived ASCs FC >0.9. Most of the regulated genes are shared between groups, but have been assigned to the primary group for the sake of clarity. NS, not significant.

**Table 3:** **Expression levels for regulated genes according to primary gene ontology function**

		**Serum-deprived**			**VEGF-stimulated**		
**Gene ID**	**Gene name**	**FC**	**2** ^**|FC|**^		**FC**	**2** ^**|FC|**^	
***Proliferation***							
*SPRY1*	Sprouty homolog 1, antagonist of FGF signaling	+1.493		+2.815	NS		NS
*TEK*	TEK tyrosine kinase, endothelial	+1.024		+2.033	NS		NS
*JAG1*	Jagged 1	+0.8085		+1.751	+0.9621		*+1.948*
*CDKN3*	Cyclin-dependent kinase inhibitor 3	−1.668		−3.178	−1.386		−2.614
*MKI67*	Antigen identified by monoclonal antibody Ki-67	NS		NS	−1.222		−2.333
*CCNB1*	Cyclin B1	−1.324		−2.504	NS		NS
*HMOX1*	Heme oxygenase (decycling) 1	−1.047		−2.066	*−0.969*		*−1.957*
*TPX2*	TPX2, microtubule-associated	NS		NS	−1.042		−2.058
***Secretome***							
*IGF1*	Insulin-like growth factor 1	+1.963		+3.897	+1.999		+3.998
*BMP6*	Bone morphogenetic protein 6	+1.902		+3.738	+1.980		+3.944
*TRIL*	TLR4 interactor with leucine-rich repeats	+1.691		+3.229	+2.005		+4.013
*PCSK5*	Proprotein convertase subtilisin/kexin type 5	+1.091		+2.131	+1.480		+2.791
*FGF9*	Fibroblast growth factor 9	+1.044		+2.061	*+0.871*		*+1.829*
*HGF*	Hepatocyte growth factor	NS		NS	+0,9719		+1,961
*PDGFD*	Platelet derived growth factor D	+1.048		+2.067	*+0.900*		*+1.866*
*GREM1*	Gremlin 1, DAN family BMP antagonist	−0.960		−1.946	−0.918		−1.891
*TGF1*	Transforming growth factor beta1	−0.962		−1.965	−0.908		−1.877
*MME*	Membrane metallo-endopeptidase	−1.322		−2.500	−1.432		−2.699
*GREM2*	Gremlin 2, DAN family BMP antagonist	−2.326		−5.019	−2.425		−5.368

Pre-defined groups. Fold changes (FC) and consequently calculated amount of up (+) or down (−) regulation (2^|FC|^) of significant genes (*P* < 0.05) compared to adipose tissue-derived stromal cells (ASCs) in complete medium are listed for vascular endothelial growth factor (VEGF)-treated and serum-deprived ASCs FC >0. 9. NS, not significant.

Selected genes were up to 5.5 times upregulated. A 5.5-times upregulation was calculated as 2 to the power of the significant fold change (2^FC^). Hence, a 5.5-times upregulation was derived from a positive fold change of 2.4564 equal to 2^2.4564^. The following results are presented as x times up- or downregulations based on calculated log2 measures.

### Angiogenesis

The angiogenesis category proved to have only up- or downregulated single genes that were shared by other chosen categories according to GO terms. Bone morphogenetic protein 6 (*BMP6*) was found to be nearly four times upregulated for both serum-deprived and VEGF-stimulated ASCs compared to ASCs from complete medium. Fibroblast growth factor 9 (*FGF9*) was also significantly upregulated times two for the serum-deprived ASCs as well as VEGF-stimulated ASCs, while Integrin beta 3 (*ITGB3*) was two times downregulated in both.

### Adhesion

A single gene in the adhesion category was significantly upregulated for both comparison groups versus control. That gene was Calsyntenin 2 (*CLSTN2*), which showed a more than five times increase in expression. Junctional adhesion molecule B (*JAM2*) was two times significantly upregulated for serum-deprived cultures. Podocalyxin-like protein 1 (*PODX*) and ITGB3 were approximately two times downregulated with serum deprivation as well as VEGF stimulation (Table 2).

### Extracellular matrix

SPARC related modular calcium binding 2 (*SMOC2*) is the highest upregulated gene in the extracellular matrix (ECM) category, corresponding to 4.2 and 4.5 times, respectively, in the serum-deprived and VEGF-stimulated groups compared to control. Spondin 1/F-spondin (*SPON1*) was two times upregulated in serum-deprived cultures and three times upregulated in VEGF-stimulated cultures. ADAM metallopeptidase with thrombospondin type 1 motif (*ADAMTS12*), sarcoglycan delta (*SGCD*), dermatopontin (DPT) and microfibrillar-associated protein 4 (*MFAP4*) were approximately two times upregulated in both conditions. Protein tyrosine phosphatase receptor type B (*PTPRP*) was significantly downregulated in VEGF-stimulated cultures only, as opposed to transglutaminase 2 (*TGM2*) which was significantly downregulated in serum-deprived cultures only. Matrix metallopeptidase 1 and 3 (MMP1 and MMP3) were downregulated 4.4 and 2.8 times, respectively, significantly only for serum-deprived cultures. Transcription of collagen 13 alpha 1 (*COL13A*) was also downregulated (Table 2).

### Secretome

The secretome category presented five upregulated growth factor genes: insulin-like growth factor 1 (*IGF1*), *BMP6*, *FGF9*, hepatocyte growth factor (*HGF*) and platelet-derived growth factor D (*PDGFD*). They were all upregulated two to three times in both conditions, except for HGF being only significantly upregulated in VEGF-stimulated cultures. The growth factor transforming growth factor beta1 (*TGFB1*) was two times downregulated. TLR4 interactor with leucine-rich repeats (*TRIL*) was upregulated three times for serum-deprived cultures and four times for VEGF-stimulated cultures. Proprotein convertase subtilisin/kexin type 5 (*PCSK5*) was 2.1 and 2.8 times upregulated for the serum-deprived and VEGF-stimulated groups. Gremlin 1 and 2, DAN family of BMP antagonists (*GREM1* and *GREM2*) were both downregulated, GREM2 most substantially with 5.0 and 5.4 times downregulation in serum-deprived and VEGF-stimulated cultures, respectively. Membrane metallo-endopeptidase (MME) was downregulated two times (Table 3).

### Proliferation

Overall, three genes were significantly upregulated in the GO-defined proliferation category and four genes were significantly downregulated. Unique to the proliferation category was sprouty homolog 1, antagonist of FGF signaling (*SPRY1*) upregulated 2.8 times in serum-deprived ASCs. TEK tyrosine kinase, endothelial (*TEK*) was two times upregulated, also only significantly for serum-deprived ASCs. Jagged-1 (*JAG1*) was significantly upregulated in both conditions 1.7 and 1.9 times, respectively. In this category, cyclin-dependent kinase inhibitor 3 (*CDKN3*) was downregulated 3.2 times (serum-deprived ASCs) and 2.6 times (VEGF-stimulated ASCs), along with antigen identified by monoclonal antibody Ki-67 (*MKI167*), cyclin B1 (*CCNB1*), heme oxygenase (decycling) 1 (*HMOX1*) and *TPX2*, microtubule-associated (*TPX2*) (all downregulated approximately two times; for MKI67 and CCNB1 only significantly so for VEGF-stimulated and serum-deprived cultures, respectively (Table 3)).

### Confirmational quantitative PCR

**Figure 2:**
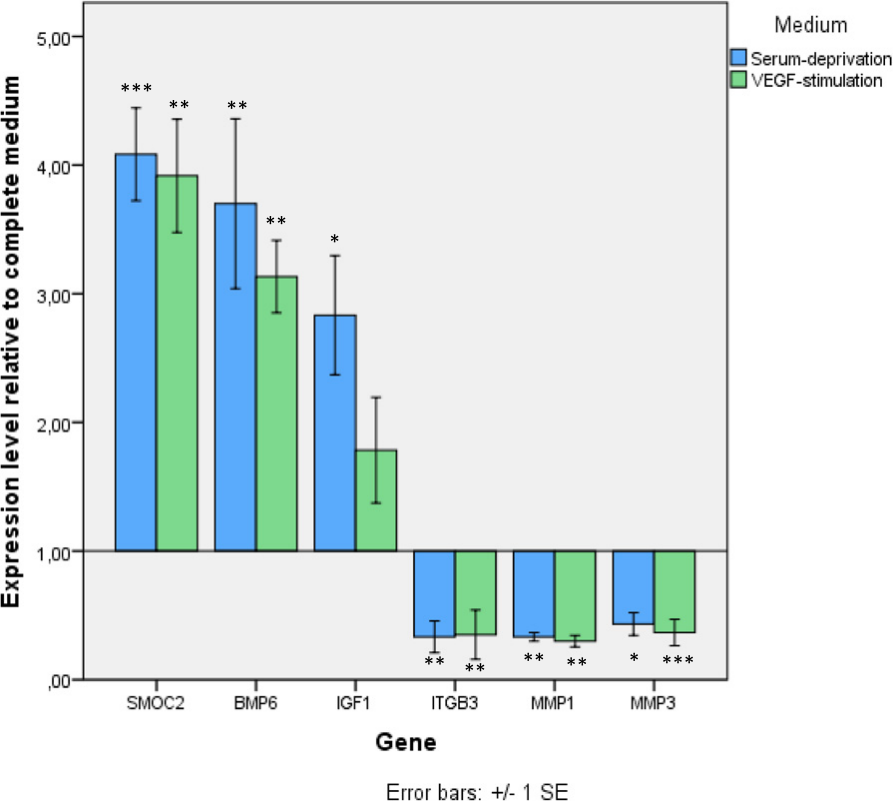
mRNA expression levels by quantitative PCR for selected genes of interest. Quantitative PCR results for three up- and downregulated genes of vascular endothelial growth factor (VEGF)-stimulated or serum-deprived adipose tissue-derived stromal cells compared to control correspond with significant microarray data. ***P < 0.001, **P < 0.01, **P < 0.05. N = 3. BMP6, Bone-morphogenic protein; IGF1, insulin-like growth factor 1; ITGB3, integrin-beta 3; MMP, Matrix metalloproteinase; SE, standard error of the mean; SMOC2, SPARC-related modular calcium binding 2.

Quantitative PCR results confirmed microarray analysis (Figure 2). *SMOC2* was shown to be four-fold upregulated by both methods for VEGF-stimulated or serum-deprived ASCs compared to control. *BMP6* was 3.7 and 3.2 times upregulated on quantitative PCR for serum-deprived and VEGF-stimulated ASCs compared to 3.7 and 3.9 times, respectively, on microarray analysis. Results for *IGF1* were less consistent with 2.8 (serum-deprived ASCs) and 1.8 (VEGF-stimulated ASCs) times upregulation on quantitative PCR with nearly four times upregulation for both groups on microarray. The *ITGB3* 2.5 times downregulation by serum deprivation or VEGF stimulation on quantitative PCR confirmed the two times downregulation of this gene on microarray. *MMP3* was shown to be downregulated 3.3 times by quantitative PCR and 2.8 times by microarray analysis, while *MMP1* was also downregulated in both analyses but not to the same extent observed with quantitative PCR (3.3 times for both groups) as with microarray (4.4 times).

## Discussion

ASCs have therapeutic regenerative potential for patients with ischaemic heart disease as well as for multiple other degenerative diseases. *In vitro* preconditioning of ASCs with VEGF-stimulation and serum-deprivation prior to administration is applied pursuing enhanced efficacy of treatment in an angiogenic direction. VEGF stimulation is believed to promote angiogenesis, but regenerative molecular mechanisms are numerous and current comparative analysis of the gene profile of VEGF-stimulated and serum-deprived ASCs have revealed other potentially contributing factors. Gene Enrichment Analysis linked with KEGG library and GO terms point towards a large regulation of cellular components involved in the extracellular space and thereby cell-to-cell or cell-to-ECM communication. These include cytokines and growth factors, adhesion molecules and a variety of ECM constituents.

Surprisingly, our data showed that serum deprivation of ASCs rather than VEGF stimulation was the decisive factor for the observed change in ASC gene expression *in vitro*.

Interpreting this observation, the contribution from endogenous VEGF production is a potential confounder in case the endogenous production exceeds the dose used during VEGF stimulation. Our laboratory has identified that the endogenous production of VEGF from ASCs in the present protocol does take place (unpublished data). Microarray analyses showed that transcription of VEGF was significantly upregulated with serum deprivation and VEGF stimulation in the present study. Fold change was below our cut-off value and was therefore not considered (fold change 0.35; *P* < 0.04), but it pointed out that endogenous VEGF production did take place and was stimulated by serum starvation, as in agreement with observations made by Chua and colleagues [16]. The production of endogenous VEGF in our ASC cultures is, however, in the lower picogram/ml range and is therefore considered to play an insignificant role compared to the 50 ng/ml exogenous VEGF added during VEGF stimulation.

The present microarray analyses do not reveal the level of VEGF receptors in our ASC cultures, nor do they reveal receptor ligand binding. We merely conclude that test circumstances do not significantly change the transcription of VEGF receptors. In other words, results do not explain why VEGF is not decisive. However, as previously discussed by Follin and colleagues [8] and Chua and colleagues [16], we suggest that VEGF needs to be part of a more complex stimulation protocol in order to exercise sufficient effectiveness.

Serum deprivation proves to have an impact on the ASC secretome and interaction with the extracellular environment. We identified a significant upregulation of growth factors *IGF1* (nearly four times upregulated), *PDGFD* and *FGF9* (two times) which corresponds with other microarray studies performed on bone marrow-derived stem cells where *IGF1* was highly upregulated in long-term serum-deprived cells [17]. IGF-1 secreted from ASCs is known to initiate cardioprotective actions inhibiting cardiomyocyte apoptosis *in vitro* as well as *in vivo* [18,19]. FGF9 (also known as heparin binding growth factor HBGF) is a member of the fibroblast growth factor family. FGF family members possess broad cell survival activities, and are involved in a variety of biological processes, including morphogenesis and tissue repair [20]. Platelet-derived growth factor, PDGFD, a newly recognised member of the PDGF family, has recently been shown to promote fibrogenesis of cardiac fibroblasts. PDGFD elevates cardiac fibroblast proliferation, myofibroblast differentiation, and type I collagen secretion; it regulates matrix metalloproteinase activities and TGF-β pathways and has a significant effect on ECM turn-over [21].

We also found that *BMP6* was nearly four times upregulated in conditioned ASCs and we identified a five times downregulation of *GREM2*, a BMP-6 antagonist. BMP-6, along with the other members of the BMP family, has diverse biological activities in various systems, generally stimulating differentiation and inhibiting proliferation. In concert, BMP6 and GREM2 play roles in organogenesis and tissue differentiation. During embryonic development, GREM2 is required for atrial differentiation along with establishment of cardiac rhythm [22]. The blocking of BMP inhibits differentiation and promotes the expansion of stem cells [23].

It is empirical that serum starvation inhibits proliferation in cell cultures. In fact, the rationale behind the used cultivation protocol is that serum starvation combined with VEGF stimulation favours MSC and ASC endothelial differentiation at the expense of proliferation [2,4]. In a previous publication we showed that ASC differentiation towards endothelium requires a more complex protocol than serum deprivation combined with VEGF stimulation only [8]. In the present study we hypothesised that the present cultivation protocol would favour secretion of pro-angiogenic factors and thereby stimulate angiogenesis by paracrine mechanisms. GO analyses of microarray data, however, do not show any direct augmented pro-angiogenic paracrine activity as a consequence of the used protocol. The observed increase in *BMP6* production does correlate with the desired inhibition of proliferation; however, BMP6 production is known for its involvement in cardiovascular development stimulating cardiomyocyte differentiation rather than endothelial [24]. Stimulation of cardiomyocytes is further supported by the observed upregulation in the protein Jagged-1. Jagged-1 is involved in cell-cell signalling, and murine MSCs are known to promote proliferation of cardiomyocytes by Jagged1:Notch1 signalling [25].

In concert the observed changes in the ASC secretome during serum starvation suggest that used pre-stimulation protocol activates signals that favour cardiac repair by supporting cardiomyocyte protection, differentiation and proliferation, cell survival in general, and activation of fibroblasts favouring ECM turnover.

In general, changes in paracrine activity induce dynamic changes in ECM composition, regulating the inflammatory, fibrogenic, and angiogenic pathways at different stages of regeneration in the infarcted heart. Matricellular proteins induced following tissue injury associate with growth factors and cytokines, and modulate cell-cell and cell-matrix interactions. Matricellular proteins play no structural role, but modify cell function by regulating ECM assembly, adhesion, ECM protease activity and growth factor signalling, and as such are believed to play an important role in the regulation of cardiac repair [26]. Matricellular proteins promote de-adhesion, creating an intermediate cellular adhesive state that activates survival signals [27]. Matricellular proteins include the SPARC (secreted protein acidic and rich in cysteine) family and thrombospondins. SPARC has proved to be important for organisation of the scar and maturation of collagen after myocardial infarction; overexpression of SPARC protects against cardiac dilatation [28]. A member of the SPARC family has been identified as secreted modular calcium-binding protein 2 (SMOC-2) [29]. SMOC-2 potentiates the angiogenic effects of growth factors. Overexpression of SMOC-2 in endothelial cells synergises with VEGF or bFGF to stimulate DNA synthesis, migration, and tube formation [30]. Thrombospondin 1, an inhibitor of angiogenesis and cell proliferation, was downregulated in such SMOC-2-overexpressing endothelial cells. In our setting the expression of Thrombospondin 1 was insignificantly affected by the treatment and, therefore, not directly correlated with SMOC-2 expression (data not shown).

SPON1 (F-Spondin or vascular smooth muscle cell growth-promoting factor) belongs to the thrombospondins and contains the thrombospondin type 1 repeat (TSR) involved in matrix organisation and cell-cell interactions [31,32]. F-Spondin inhibits angiogenesis, namely VEGF- or bFGF-stimulated migration of HUVECs (Human Umbilical Vein Endothelial Cells), and specifically ITGB3-mediated endothelial cell spreading [33]. In turn, SPON1 stimulates proliferation of smooth muscle cells in the vascular wall. Thus, inhibition of angiogenesis is followed by the growth of smooth muscle cells and the maturation of vessels [33].

Culturing ASCs with VEGF and serum deprivation we have identified that *SMOC-2* is more than four times upregulated and *F-Spondin* is two (serum-deprivation) to three times (VEGF-stimulation) upregulated. Both proteins are involved in matrix regulation; however, possibly with opposing effects on angiogenesis and endothelial behaviour. This underscores the importance and complexity of cell-ECM interactions and the need to examine these matters more thoroughly. In concert, observations indicate that VEGF stimulation and serum deprivation does affect regeneration and angiogenesis, but indirectly so, by modulating ECM, generating or preventing a permissive environment.

*SMOC-2* has recently been identified as a stem cell marker in the intestinal crypt base cells. Switching the status of active WNT/inactive BMP pathways of human intestinal epithelial crypt cells (from the fetal intestine) triggers a conversion towards a stem cell-like state with expression of genes including *SMOC-2* [34]. MSCs cultured in hypoxic conditions (5% O_2_) display an enhanced expression of genes involved in plasticity with the most highly upregulated gene in this group being *SMOC-2* [35]. While culture in hypoxia maintains MSCs in a multipotent undifferentiated state, they express more adhesion and ECM molecules, including SMOC-2, than MSCs cultured in normoxic conditions [35]. It appears that culturing ASCs with serum deprivation mimics limited blood supply and thereby hypoxia as well as *in vivo* ischaemic conditions. The observed increase in *SMOC-2* expression suggests that our protocol favours stemness.

SPARC along with other matricellular proteins are known to reorganise actin fibres and disassembly of focal adhesion molecules to facilitate cell migration when a cell undergoes transformation from a strong to an intermediate adhesive state in response to injury or other types of tissue remodelling [27]. The soluble SPARC protein was originally found to induce MMP-1, MMP-3, and MMP-9 activity in synovial fibroblasts [36]. MMP-1 promotes collagen breakdown in the infarcted area of the heart and MMP-3 is an upstream activator of MMP-9, which can break down scar tissue and permeabilise the ECM to allow for revascularisation in an acute infarct [37,38]. Furthermore, it has been suggested that MMP inhibition protects from adverse tissue remodelling [39].

We identify a noticeable downregulation of *MMP 1* and *MMP3* in ASCs cultured with serum deprivation, indicating a stabilising effect on the ECM. In addition we find that *ADAMTS12*, a metalloproteinase with thrombospondin motifs, is two times upregulated. ADAMTS12 binds to the ECM and has been shown to sequester VEGF, leading to inhibition of endothelial proliferation [40,41]. ADAMSTS12 is upregulated in bone marrow-derived MSCs with increasing *in vitro* passages and is inversely associated with growth and proliferation in these cells [42].

Normal wound healing as well as myocardial regeneration is initiated by an inflammatory phase followed by a proliferative phase characterised by ECM deposition and angiogenesis. The maturation phase follows, represented by matrix stabilisation and vascular maturation. The observation that ASCs stimulated with VEGF and serum deprivation downregulate collagen turnover, ECM permeabilisation and thereby migration of endothelial cells and revascularisation, but stimulates smooth muscle cells in the vascular wall, indicate that the used pre-stimulation protocol promotes cells that contribute to the maturation phase.

We find pronounced upregulation in transcription of adhesion molecules Calsyntenin-2 (*CLSTN2*) and junctional adhesion molecule JAM-2. CLSTN2 belongs to the cadherins and a family of postsynaptic membrane proteins, and JAM2 has been associated with endothelial junctions and leukocyte-endothelial interactions only. The significance of these adhesion molecules with regard to ASC-mediated regeneration remains elusive, and presently only lends support to the idea that cell-cell interactions are important for ASC mechanisms of action.

Transcription of adhesion molecules such as integrin beta 3 and Podocalyxin-like protein 1 (*PODXL*, a sialomucin) was decreased, potentially affecting cell-ECM interactions. From studies with cancer cells it is known that silencing of *PODXL* reduces migration and interaction with collagen. MSCs are also known to express *PODXL*, and the level of expression has been shown to correlate with MSC clonogenicity as well as the ability to differentiate and migrate [43,44].

The observed upregulation in the transcription of Sarcoglycan delta (*SGCD*), part of the dystrophin-glycoprotein complex muscle-specific proteins exclusively expressed in the skeletal and cardiac muscles and creating a link between the cell cytoskeleton and the ECM, supports the theory that the chosen pre-stimulation protocol mimics an environment in favour of cardiomyocyte development [45].

The significantly up- and downregulated proliferative markers all reflect the observed inhibition in proliferation with the used pre-stimulation protocols. Cellular changes, including ASC proliferation, are regulated by the growth factor activated mitogen-activated protein kinase (MAPK) signalling pathway. Expression of SPRY1, which is an inhibitor of FGF signalling and thereby MAPK signalling, is upregulated in serum-deprived ASCs, correlating with the observed decrease in proliferation. In endothelial cells, upon FGF receptor and VEGF receptor activation, SPRYs are known to translocate to the plasma membrane and inhibit proliferation [46]. In addition, cyclin-dependent kinase inhibitor 3 (*CDKN3*) and Cyclin B1(*CCNB1*), which are regulatory proteins that are essential for commitment of cells to mitosis, are downregulated in pre-stimulated ASCs. In essence the observed changes in proliferation markers image our test culture circumstances where the restricted conditions without serum halt proliferation compared to control conditions.

The regenerative effects of transplanted cells are always the sum of interactions with the surrounding tissue. The full consequence of the used *in vitro* priming of cells upon delivery into a multifactorial *in vivo* environment remains to be shown. Thorough *in vitro* characterisation of the cell products used in the clinic is, however, a valuable tool for interpretation of pre-clinical and clinical results. Where the shown effects of the present pre-stimulation protocol upon ASC transcriptional activity are tangible the projected consequences of these effects upon *in vivo* delivery are at this point to be regarded as hypothesis generating.

## Conclusion

ASCs are used as an experimental regenerative therapy for patients with ischaemic heart disease to improve myocardial perfusion and to regenerate injured myocardium. Aiming to enhance the efficacy of stem cell therapy, different regimes of *in vitro* preconditioning prior to administration are examined. We have elucidated the molecular signature of ASCs that have been preconditioned with serum deprivation and VEGF stimulation. We show that serum deprivation is the decisive factor and that serum deprivation of ASCs has an important impact on the microenvironment by the ECM components and signalling molecules that they secrete. Given that the genes regulated upon serum deprivation in ASCs are primarily involved in stabilisation of ECM, fibrogenesis, inhibition of angiogenesis, maturation of vessels, differentiation and protection of cardiomyocytes, we hypothesise that these ASCs will exert their effect on cardiac repair by regulating the dynamic cellular and ECM events during the late maturation phase in infarct healing.

## Abbreviations

ASC: adipose tissue-derived stromal cell
DMEM: Dulbecco’s modified Eagle's medium
ECM: extracellular matrix
FBS: fetal bovine serum
GO: gene ontology
KEGG: Kyoto Encyclopedia of Genes and Genomes
MAPK: mitogen-activated protein kinase
MSC: mesenchymal stromal cell
PBS: phosphate-buffered saline
VEGF: vascular endothelial growth factor
